# A comparative analysis of nutritional content changes in six Chinese cuisines prepared using industrial versus traditional hand-cooked modes

**DOI:** 10.3389/fnut.2025.1567196

**Published:** 2025-03-19

**Authors:** Xuan Wang, Jun Li, Xiaomeng Wu, Sai Fan, Zhu Wang, Yunfeng Zhao, Jingguang Li, Dawei Chen

**Affiliations:** ^1^NHC Key Laboratory of Food Safety Risk Assessment, Food Safety Research Unit (2019RU014) of Chinese Academy of Medical Science, China National Center for Food Safety Risk Assessment, Beijing, China; ^2^College of Food Science and Nutritional Engineering, China Agricultural University, Beijing, China; ^3^Beijing Center for Disease Prevention and Control, Beijing, China; ^4^National Institute for Nutrition and Health, Chinese Center for Disease Control and Prevention, Beijing, China

**Keywords:** Chinese cuisine, nutritional changes, industrial cooked modes, traditional hand-cooked modes, vitamin

## Abstract

**Objective:**

With the rise of industrialized dishes, the quality differences between industrial and traditional hand-cooked modes are a major concern for the food industry and consumers. This study examined the nutrient content variations in Chinese cuisines between these two cooking modes, addressing a crucial research gap.

**Methods:**

To account for moisture changes during cooking, water content in both raw ingredients and cooked dishes was adjusted, and nutrients were reported on a dry matter basis. The changes in nutrient content pre- and post-cooking were assessed by calculating the relative percentage of nutrient content in the cooked dishes in comparison to their levels in the raw ingredients. An independent *t*-test was employed to assess the significance of differences in the changes in nutrient content between industrial and traditional hand-cooked modes.

**Results:**

Macronutrient levels remained relatively stable, with changes of less than 20% across most dishes for both cooking methods, with some exceptions noted. Fat-soluble vitamins A and D exhibited minor fluctuations, ranging from 2.6 to 39.4%, while vitamin E levels consistently increased. In contrast, water-soluble vitamins, specifically B1, B2, B3, and B6, experienced substantial decreases across all examined dishes. The fatty acid profiles were consistent with the fat content, and mineral content demonstrated a moderate increase under both cooking conditions. An inter-group *t*-test indicated no significant differences in nutrient content changes between the two cooking modes (*p* > 0.05), except for vitamin B6 retention, which was significantly lower in traditional hand-cooked modes compared to industrial modes (*p* < 0.05).

**Conclusion:**

Among the six Chinese cuisines analyzed, the changes in nutrient content did not significantly differ between industrial and traditional cooking methods, with the notable exception of vitamin B6 retention. These findings contribute to a deeper understanding of how different cooking techniques impact the nutritional value of food, providing valuable insights for dietary decision-making and food processing technologies.

## 1 Introduction

Chinese cuisine, renowned globally for its intricate flavors and diverse cooking techniques, occupies a prominent position not only domestically but also across internationally (1). Within the framework of traditional dietary cultures, artisanal cooking methods have historically played a crucial role in preserving the gustatory characteristics and nutritive quality of culinary preparations (2). However, as modern society advances, the trend toward food industrialization has become increasingly evident, prompting many individuals to adopt industrialized cooking paradigms that prioritize efficiency and uniformity.

Over the past few decades, the prevalence of industrialized culinary approaches, exemplified by rapid-processing food technologies and the rise of prepared dishes, has significantly altered consumer perceptions of dietary composition. These transformations have sparked widespread concern regarding the preservation of food’s nutritive properties (3). Industrial cooking methods exert a dual influence on the nutritional quality of food. While they may potentially cause the breakdown of heat-sensitive nutrients, they also offer opportunities to enhance the bioavailability of certain dietary components through technical refinements. An illustrative example is the application of precise temperature control technologies, such as sous-vide cooking, which has been demonstrated to increase the retention rate of *ω*-3 fatty acids in salmon by 15–20%, thereby preserving its nutritional value more effectively (4). The exposure to high temperatures and extended heating periods during industrial cooking can result in a substantial decrease in vitamin and mineral content (5). The existing body of research investigating the influence of cooking on food nutrition predominantly centers on the impacts of various cooking methods and conditions. Variations in cooking methodologies and the specific conditions employed can markedly affect the nutritional integrity of foodstuffs. For instance, moist-heat processing methods, such as steaming and boiling, are generally recognized for their superior ability to conserve water-soluble vitamins and minerals. Conversely, dry-heat cooking practices, particularly those characterized by high-temperature frying, are often associated with a pronounced nutrient depletion (6). Similar findings were reported by Li et al. (7), who investigated the influence of six distinct cooking methods on the nutrient composition, bioaccessibility, and biological activities of *Boletus auripes*. Their results revealed that boiling, steaming, and microwaving were particularly beneficial for preserving and enhancing the digestibility of nutrients within this fungal species.

Currently, research comparing dishes prepared using industrialized and traditional hand-cooked modes primarily focuses on flavor attributes and consumer preferences. In contrast to traditionally hand-cooked dishes, pre-prepared dishes, especially meat products, undergo production and/or reheating at high temperatures, which can lead to the development of warmed-over flavor due to lipid peroxidation and protein denaturation (8). The findings of Tharrey et al. (9) indicate that industrially processed meals present a more economical option for consumers when the time invested in home meal preparation is monetarily valued. Considering the increasingly demanding nature of modern life, greater consideration should be given to the implicit cost imposed on consumers by the time they dedicate to preparing meals at home. Notably, there is a significant lack of research dedicated to a detailed comparison of nutrient content modifications resulting from industrialized versus traditional hand-cooked modes in culinary preparations. Therefore, it is imperative to examine the influence of industrialized and traditional hand-cooked modes on the nutritional profile of Chinese culinary preparations. This issue not only involves in-depth research in nutritional science but also concerns the health choices of modern consumers. The present study aims to bridge the gap by providing a scientific foundation for both theoretical understanding and practical applications, thereby contributing to the advancement of knowledge tin this crucial area.

For the purposes of this study, six widely prevalent Chinese culinary preparations were selected from the market. These dishes serve as examples of traditional Chinese cooking methods, including steaming, boiling, and stir-frying. Ingredients were carefully formulated according to specified recipes, and the dishes were then prepared using both industrialized cooking protocols and traditional home-cooking practices, leading to the development of corresponding cooked products. A comparative analysis was conducted to evaluate the differences in nutrient alterations between industrialized and traditional home-cooking modes, by examining the changes in various nutrients both before and after the cooking process.

## 2 Materials and methods

### 2.1 Materials and reagents

The selection of the six industrially produced Chinese cuisines was made according to consumer preferences, as indicated by sales data collected from major online sales platforms in China. These dishes specifically include Braised Pork in Brown Sauce (BPBS), Braised Beef with Radish (BBR), Steamed Pork with Preserved Vegetables (SPPV), Braised Tomato Beef (BTB), Braised Beef with Potatoes (BBP), and Braised Pork Ball in Brown Sauce (BPBBS). In accordance with the specified recipe for each dish, the appropriate raw materials and condiments were gathered. These ingredients and seasonings were then prepared in their pre-cooking state, thoroughly combined, and apportioned into three equal parts. One part was retained as an uncooked control sample, whereas the remaining two parts were allocated for preparation using industrialized cooking methods and traditional home-cooking methods, respectively. In this study, all standard substances and enzymes utilized were procured from Sigma-Aldrich. The analytical-grade extraction reagents employed were acquired from Sinopharm Chemical Reagent Co., Ltd.

### 2.2 Cooking methods

The preparation procedure for Braised Pork Ball in Brown Sauce (BPBBS) followed the standard DB32/T 1548-2009, titled “General Specifications for Huaiyang Cuisine (10).” The dishes Braised Pork in Brown Sauce (BPBS) and Braised Beef with Potatoes (BBP) were processed according to DB51/T 1728-2014, “Cooking Process Specifications for Chinese Sichuan Cuisine (11).” Meanwhile, the dishes Braised Beef with Radish (BBR), Steamed Pork with Preserved Vegetables (SPPV), and Braised Tomato Beef (BTB) were prepared based on DB4401/T 38–2020, “Cooking Process Specifications for Guangdong Regional Flavor Dishes (12).” Specific preparation methods for each cuisine were provided in Supplementary material.

### 2.3 Measurement of macronutrients

Moisture content and ash was measured as described by moisture meter (Mettler Toledo HE53, Switzerland) (120°C) and a muffle furnace (Nabertherm LV9, Germany) (550°C) (13). Crude protein contents of the dishes were determined by multiplying N contents by factor 6.25 (14). Total fat content was determined after acid hydrolysis and solvent extraction using a Diethyl ether (15). The cholesterol content was quantified following the methodology outlined by Wu et al. (16). In summary, the samples underwent saponification using an ethanol-potassium hydroxide solution, followed by extraction with petroleum ether and anhydrous ether. The extracts were then concentrated, dissolved in ethanol, and diluted prior to analysis. High-performance liquid chromatography (HPLC) (Waters e2695, USA) was employed for quantification, utilizing the external standard method. The dietary fiber content was assessed in accordance with AOAC Method 991.43 (17). In brief, the samples were subjected to defatting, desugaring, drying, crushing, and sieving. Subsequently, they were digested with heat-stable *α*-amylase, protease, and amyloglucosidase. The resulting enzyme hydrolysate was precipitated using ethanol, filtered, and the residue was washed with ethanol and acetone. The total dietary fiber (TDF) content was determined by drying and weighing the residue, and subtracting the masses of protein, ash, and reagent blank from it. The total sugar was determined by the DNS method (18). Quality control (QC) samples were incorporated to evaluate the reliability of the assay, with each measurement conducted in triplicate.

### 2.4 Measurement of vitamins

Thiamine (Vitamin B1) contents of the samples were determined using the same extraction and chromatographic method as described by Tuncel et al. (19). Briefly, the samples were subjected to constant temperature acid hydrolysis in a dilute hydrochloric acid medium, followed by neutralization and enzymatic hydrolysis. The resulting hydrolysate was then derivatized with an alkaline potassium ferricyanide solution, extracted with n-butanol, separated using a C18 reverse-phase chromatography column, and HPLC (Waters e2695, USA) with a fluorescence detector (Waters 2,475, USA), employing an external standard method for quantification. Riboflavin (Vitamin B2), nicotinic acid (Vitamin B3) and pyridoxine (Vitamin B6) were determined by adopting the procedure described by AOAC (960.46) (20). To summarize, the samples are first subjected to pre-treatment steps including enzymatic hydrolysis and protein precipitation. Following this, they are extracted via ultrasonic oscillation under weakly acidic conditions. The resulting extracts are separated using a C18 column (1.7 μm, 2.1 mm × 150 mm) and detected by a PAD detector (Waters 2,998, USA). Quantification is achieved using the external standard method. Vitamin A, D and E were determined using HPLC (Waters e2695, USA) after alkaline saponification (21). In brief, following saponification with a potassium hydroxide ethanol solution, the sample undergoes liquid–liquid extraction to isolate and purify the fat-soluble vitamins from the saponified solution. One-dimensional liquid chromatography (1D-LC) employs a PFP column to separate retinol, vitamins D (D2 and D3), *α*-tocopherol, α-tocotrienol, *β*-tocopherol, *γ*-tocopherol, and *δ*-tocopherol from other impurities. Subsequently, vitamins D (D2 and D3) are transferred into a two-dimensional liquid chromatography (2D-LC) system through column switching techniques. Within this 2D-LC system, vitamins D2 and D3 are further separated using a PAH column. Detection in the 1D-LC is achieved using a fluorescence detector (Waters 2,475, USA) for retinol, vitamins D (D2 and D3), *α*-tocopherol, α-tocotrienol, *β*-tocopherol, *γ*-tocopherol, and *δ*-tocopherol, while detection of vitamins D2 and D3 in the 2D-LC is accomplished using a PAD detector (Waters 2,998, USA). Quantification is conducted using the external standard method. QC samples were incorporated to evaluate the reliability of the assay, with each measurement conducted in triplicate.

### 2.5 Measurement of fatty acid

The fatty acids containing free and bonded fatty acids were quantified by hydrolysis with HCl (8 M), extraction with anhydrous ether, and solvent removal. Subsequently, methyl esterification of the extracted crude fat (50 mg) was performed by adding methanol (2 mL) and sulfuric acid (200 μL) and incubating at 37°C for 30 min. n-Heptane (2 mL) was then added to extract the esterification products for determination by GC (Agilent 7890A, USA) equipped with a flame ionization detector (FID) and Agilent CP-Sil88 capillary column (100 m × 0.25 mm I.D. × 0.2 μm film thickness). The column temperature procedure was as follows: The initial oven temperature was 120°C, maintained for 10 min, then raised at a rate of 3°C /min up to 180°C, then raised at a rate of 1.5°C /min up to 200°C for 3 min, and then raised at a rate of 2°C /min up to 225°C for 20 min. The carrier gas was helium (purity>99.999%) with the flow rate of 1.3 mL/min. The column head pressure was set as 38 psi, the split ratio was 30:1, and the injection volume was 1.0 μL (22). QC samples were incorporated to evaluate the reliability of the assay, with each measurement conducted in triplicate.

### 2.6 Measurement of minerals

The sample before digestion (1 g) and simulated digestion supernatant (1 mL) were weighed into a 50 mL polytetrafluoroethylene canister. Aqua regia (5 mL) was then added, and the mixtures were incubated for 15 min at room temperature. These were sealed and placed into the microwave digestion system with continuous heating at 240°C for 2 h for digestion, and insisting on heating to evaporate acid to 2 mL after digestion. Subsequently, the digestion beakers were removed and cooled to room temperature. The digestion solutions were transferred into a volumetric flask and diluted to 10 mL with deionized water. An empty digestion vessel without sample addition was used as a blank. The contents of the eight elements were measured using inductively coupled plasma mass spectrometer (ICP-OES, Agilent 720ES, USA) (7). QC samples were incorporated to evaluate the reliability of the assay, with each measurement conducted in triplicate.

### 2.7 Statistical analysis

To eliminate compositional alterations resulting from changes in moisture content during the cooking process, the water content of both the raw ingredients and the post-cooking dishes was corrected. Nutrient contents are subsequently reported on a dry matter basis. The changes in nutrient content pre- and post-cooking were assessed by calculating the relative percentage of nutrient content in the cooked dishes in comparison to their levels in the raw ingredients. An independent *t*-test was employed to assess the significance of differences in the changes in nutrient content between industrial and traditional hand-cooked modes. All numerical variables were expressed as the mean and standard deviation (mean ± SD). Statistical significance and significant differences (*p* < 0.05) were evaluated by *t*-test using IBM SPSS Statistics 27.

## 3 Results and discussion

### 3.1 Changes in macronutrients

This study utilized a controlled experimental approach to examine the similarities and differences in nutrient content alterations between industrially prepared and home-cooked dishes. The change in nutrient content was quantified using the percentage ratio of the dry-weight nutrient content of the finished dishes to that of the raw ingredients. Each dish was prepared using two distinct cooking modes, resulting in two sets of nutrient content change data. One dataset represented the nutrient content changes in six dishes prepared using industrial cooking techniques, while the other represented changes in six dishes prepared using traditional home-cooking modes. The groups comparisons were conducted with an independent *t*-test to determine whether there were significant differences in nutrient content changes between the two cooking modes. This approach effectively minimizes the influence of variations in moisture content, processing techniques, and the compositional characteristics of the dishes, ensuring more accurate comparison.

**Figure 1:**
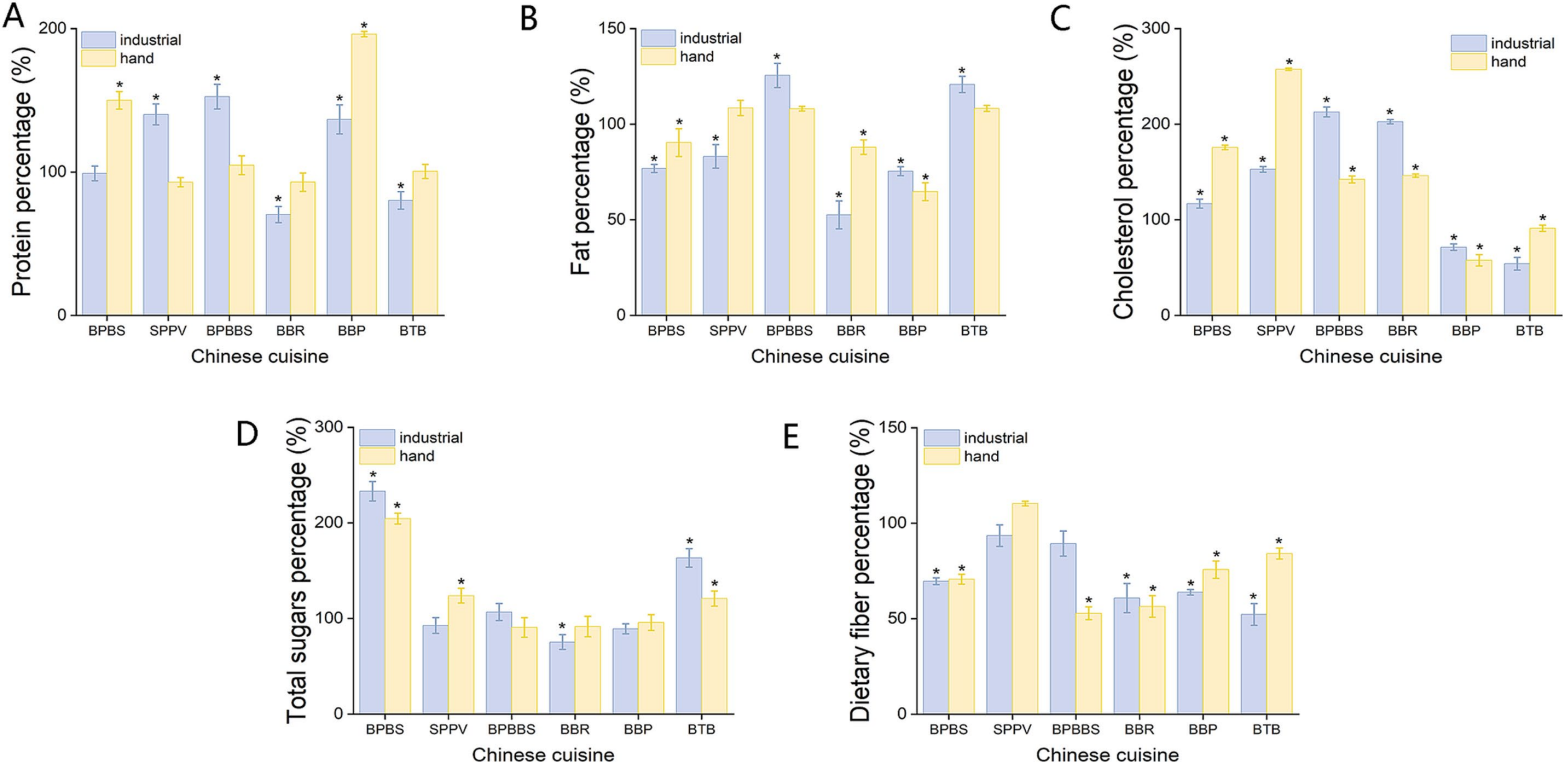
The percentage of dry-weight macronutrient content in six dishes relative to their raw materials after cooking under different cooking modes. **(A)** Protein content percentage. **(B)** Fat content percentage. **(C)** Total sugars content percentage. **(D)** Dietary fiber content percentage. **(E)** Cholesterol content percentage. *Represents that the nutrient content in the cooked dishes is significantly higher or lower than that in the uncooked raw materials (*p* < 0.05).

As illustrated in Figure 1, there is a discernible alteration in the protein content of the dishes prepared using both cooking modes. Specifically, within the industrial cooking mode, the protein content in BBR and BTB showed a reduction, ranging from 0.9 to 29.7%. In contrast, the protein content of the other four groups of dishes increased, with elevations varying from 36.7 to 52.6%. Conversely, within the traditional home-cooking mode, the changes in protein content observed in SPPV, BPBBS, BBR, and BTB were limited to no more than 10%, while the protein content in BBP demonstrated a notable elevation. Statistical analysis, conducted using a *t*-test with inter-group correction, indicated that the differences in protein content changes between the industrialized and traditional home-cooking modes did not reach statistical significance (*p* > 0.05). The primary impact of cooking methods on proteins is denaturation, which enhances protein digestibility. However, protein denaturation does not necessarily lead to a reduction in protein content (23). The observed decrease in protein content in the samples may be attributed to the hydrolysis of protein segments, as well as the solubilization and leaching of nitrogenous compounds. Furthermore, some studies have indicated that specific cooking techniques may actually increase protein content, possibly due to enzyme-mediated hydrolysis that releases free amino acids (24).

Notably, obvious changes in fat content were observed only in BTB when subjected to the industrialized cooking method, ranging from 8.2 to 25.6% (Figure 1B). For the remaining dishes, there was minimal variation in fat content between the two cooking modes, suggesting that the processing techniques had a negligible impact on their lipid profiles. Several factors during the cooking process can introduce variability and potentially influence the measured levels of fat content, including the addition of extra oil, the oxidation of fats due to thermal exposure, and the uniformity of sample preparation. These factors can affect the final fat content to varying degrees, potentially leading to discrepancies in the measured values. In terms of cholesterol, which is a lipid component predominantly found in food in a bound or conjugated form. This study revealed that dishes mainly consisting of animal-based ingredients exhibited an upward trend in cholesterol content compared to unprocessed raw materials. This finding implies that appropriate thermal processing may enhance the release of cholesterol from its bound form, potentially due to the increased cellular wall disruption caused by cooking (25). The *t*-test significance analysis revealed no statistically significant differences in the alterations of fat and cholesterol content between dishes prepared using industrial cooking modes and those prepared using traditional hand-cooking modes (*p* > 0.05).

The total sugar content in the six dishes showed minimal changes under the two cooking modes, with variation ranges remaining within 10%, except for BPBS. In the case of BPBS, the sugar content increased by more than two-fold after cooking under both cooking modes, potentially due to the addition of sugar during the cooking process for browning effects (Figure 1D). Nevertheless, based on the results of an independent *t*-test, no statistically significant differences were found in the changes of sugar content between the two groups of dishes (*p* > 0.05). After exposing the six dishes to both cooking modes, a decrease in dietary fiber content was observed across all samples to some extent, ranging from 6.5 to 47.8%. During the cooking process, exposure to elevated temperatures can compromise the structural integrity of dietary fiber, leading to its degradation into smaller molecules such as cellobiose and glucose, thereby diminishing the overall dietary fiber content. In water-based cooking methods, such as boiling and steaming, certain soluble dietary fibers, including pectin and *β*-glucan, may dissolve in the cooking water and be subsequently lost when the liquid is discarded (26). Furthermore, the cooking process disrupts plant cell walls, exposing intracellular starches that undergo gelatinization. This results in a reduction of fiber components initially classified as resistant starch (RS-1), as these starches are no longer encapsulated within intact plant cells (27). Similarly, the analysis did not reveal any significant differences in dietary fiber content between the two cooking methods (*p* > 0.05).

### 3.2 Changes in vitamins

**Figure 2:**
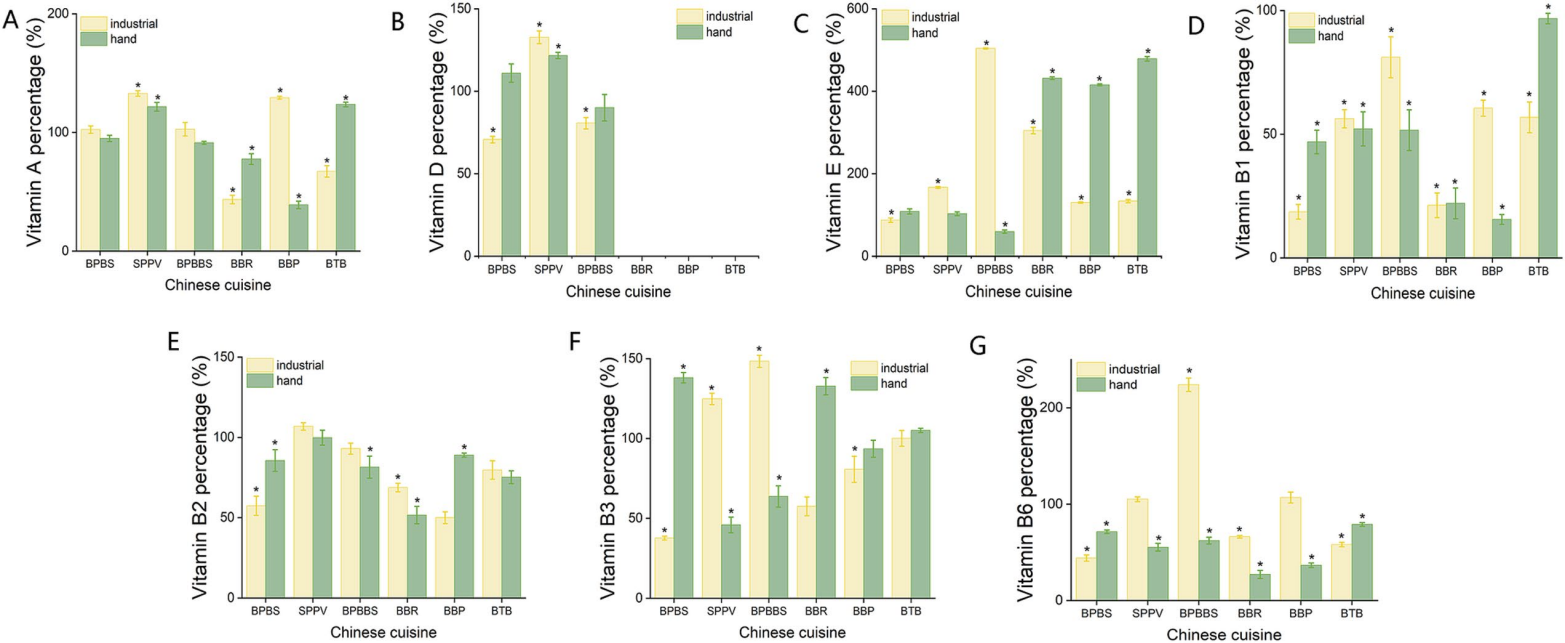
The percentage of dry-weight macronutrient content in six dishes relative to their raw materials after cooking under different cooking modes. **(A)** Vitamin B1 content percentage. **(B)** Vitamin B2 content percentage. **(C)** Vitamin B3 content percentage. **(D)** Vitamin B6 content percentage. **(E)** Vitamin A content percentage. **(F)** Vitamin D content percentage. **(G)** Vitamin E content percentage. *Represents that the nutrient content in the cooked dishes is significantly higher or lower than that in the uncooked raw materials (*p* < 0.05).

Vitamins primarily originate from the inherent composition of the ingredients and are highly susceptible to degradation upon exposure to light, heat, and oxygen. Therefore, retaining vitamin content is a crucial parameter for evaluating the suitability and effectiveness of various cooking methods. As depicted in Figure 2, across both cooking modes, the levels of fat-soluble vitamins A and D in most dishes changed minimally (ranging from 2.6 to 39.4%), whereas vitamin E levels consistently increased. The results indicate that, aside from a few specific dishes where the vitamin levels slightly decreased or demonstrated an increasing trend, the content of these vitamins generally remained consistent across both cooking methodologies. The reduction in fat-soluble vitamin content is primarily due to the oxidation reactions caused by thermal exposure (28). The observed increase in tocopherol content in certain dishes may be attributable to the addition of cooking oils rich in tocopherols or the accelerated solubilization and release of lipophilic components during cooking (25). Statistical analysis using an inter-group *t*-test revealed no significant differences in fat-soluble vitamin content between the two cooking modes (*p* > 0.05).

After exposure to both cooking modes, the water-soluble vitamins (B1, B2, B3, B6) in the six dishes exhibited a decline trend, with varying degrees of reduction among the different vitamins. This decline is attributed to their low thermal stability, making them susceptible to degradation from light exposure and oxidative processes. Furthermore, their high solubility in water can lead to leaching into the cooking liquid, especially during methods involving prolonged water contact, such as stewing or steaming (29). Notably, across both cooking modes, the reduction in the levels of vitamins B2 and B3 in most dishes was generally less substantial than that observed for vitamin B1. This stability is due to niacin and niacinamide being the most stable members of the B family vitamin, remaining stable in the presence of air, light, and within the typical pH range of foods. They also exhibit heat resistance over a certain period, resulting in minimal degradation during cooking, storage, and canning processes (30). Vitamin B2 is prone to photodegradation when exposed to light and is unstable in alkaline environments but demonstrates stability in acidic conditions and shows good resistance to heat. Consequently, during home cooking and commercial canning processes, the losses of vitamin B2 tend to be relatively low (31). Inter-group comparative analysis revealed that only vitamin B6 showed statistically significant differences between the groups. Specifically, the retention rate of vitamin B6 in dishes prepared through home cooking was found to be significantly lower compared to those cooked using industrial modes (*p* < 0.05). This outcome is likely attributable to the application of electromagnetic heating in industrial cooking equipment. Electromagnetic heating interacts directly with food molecules through an electromagnetic field, thereby enhancing thermal conductivity efficiency and significantly reducing heating time. Consequently, the potential degradation of heat-sensitive nutrients is effectively minimized during the cooking process (26). However, given the limitations posed by the limited number of samples, the results presented herein should be interpreted with caution and require further validation to robustly confirm the observed differences.

### 3.3 Changes in fatty acids

**Figure 3:**
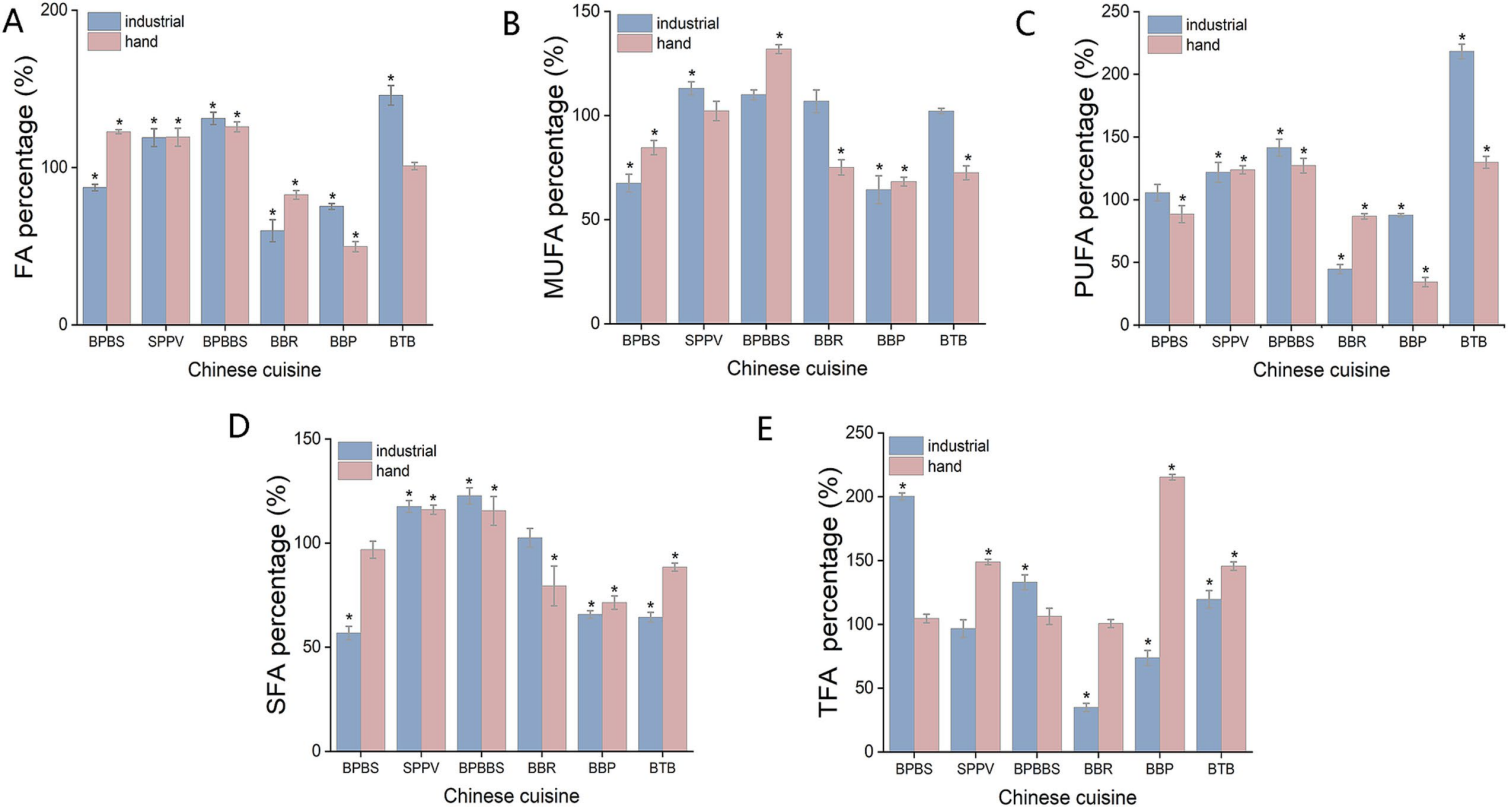
The percentage of dry-weight macronutrient content in six dishes relative to their raw materials after cooking under different cooking modes. **(A)** FA content percentage. **(B)** MUFA content percentage. **(C)** PUFA content percentage. **(D)** SFA content percentage. **(E)** TFA content percentage. *Represents that the nutrient content in the cooked dishes is significantly higher or lower than that in the uncooked raw materials (*p* < 0.05).

During the industrial cooking mode, a notable decrease in the fatty acids (FAs) content ranging from 12.7 to 40.2% was observed in dishes such as BPBS, BBR, and BTB (Figure 3). Conversely, the FAs content in dishes such as SPPV, BPBBS, and BTB increased by 18.9 to 45.9% after cooking. In terms of fatty acid composition, the changes in the saturated fatty acids (SFAs), monounsaturated fatty acids (MUFAs), polyunsaturated fatty acids (PUFAs), and trans fatty acids (TFAs) in the six dishes generally followed the same pattern as the overall changes in FA content after industrial cooking. When considering home cooking methods, the FA content of radish-stewed beef short rib and potato-braised beef decreased by 17.4 and 50.3%, respectively. Meanwhile, the FA content in the other four dishes exhibited an increase ranging from 0.9 to 25.9%. Similarly, the variations in the levels of SFAs, MUFAs, PUFAs, and TFAs in the six dishes were largely in agreement with the overall alterations in total TFA content following industrial cooking. Moreover, the fatty acid ratios and distribution in the 6 dishes were found to be generally consistent across both cooking modes. The reduction in fatty acid content can be attributed to the thermal oxidation of fatty acids. Typically, PUFAs in food are prone to instability when exposed to high temperatures, leading to their oxidation and subsequent decomposition into SFAs and MUFAs (32). Statistical analysis using an independent *t*-test revealed no significant differences between the two cooking modes (*p* > 0.05).

### 3.4 Changes in minerals

**Figure 4:**
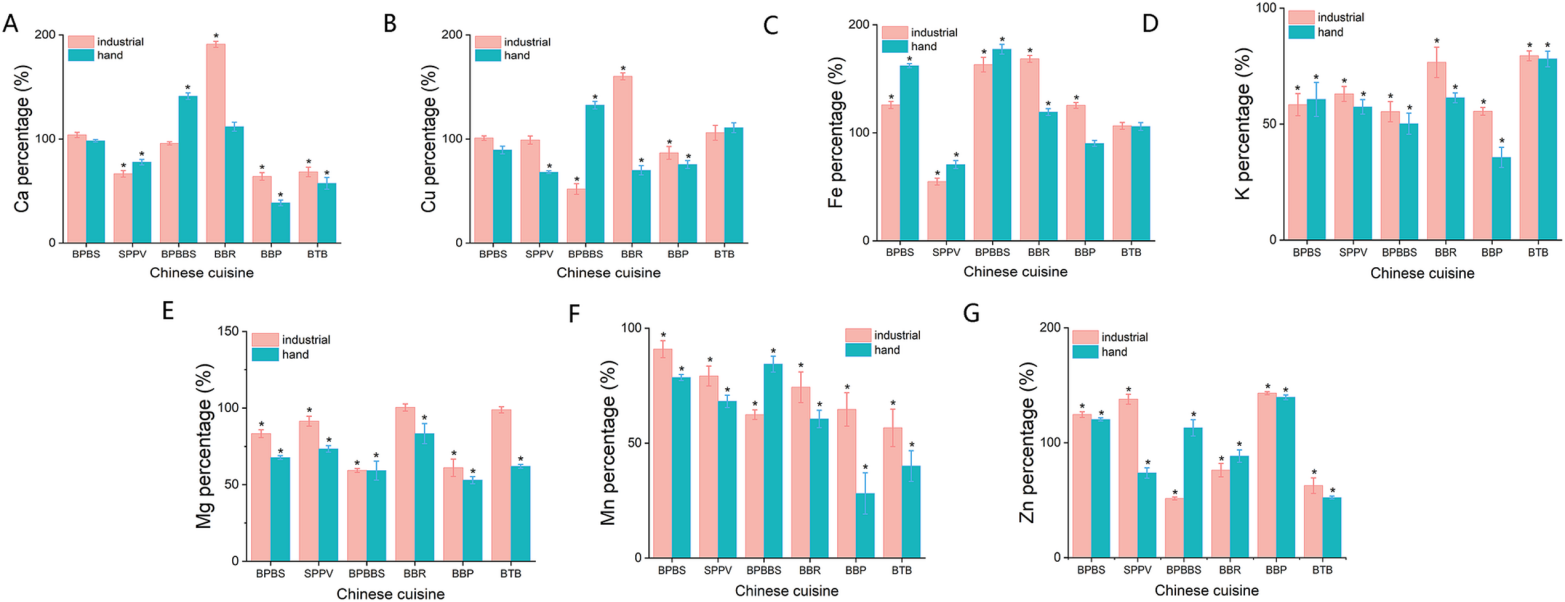
The percentage of dry-weight macronutrient content in six dishes relative to their raw materials after cooking under different cooking modes. **(A)** Ca content percentage. **(B)** Cu content percentage. **(C)** Fe content percentage. **(D)** K content percentage. **(E)** Mg content percentage. **(F)** Mn content percentage. **(G)** Zn content percentage. *Represents that the nutrient content in the cooked dishes is significantly higher or lower than that in the uncooked raw materials (*p* < 0.05).

Figure 4 illustrates the variations in the levels of seven specific minerals in the six dishes across the two different cooking modes, with each mineral showing distinct changes after cooking. In the industrial cooking method, the majority of the dishes, excluding BPBBS, exhibited an upward trend in the levels of Mg, K, Ca, Fe, Cu, Mn and Zn, with the extent of increase varying among the different minerals. This finding may be linked to the specific cooking techniques employed for different dishes. For example, BPBBS were prepared using a steaming method, which could cause certain elements to leach into the cooking water and settle at the bottom of the pot. Since the broth was not fully recoverable, this leaching process could lead to a reduction in the elemental content of the final dish. Conversely, the other dishes were prepared using a stewing method, in which the heating process could lead to the leaching of certain elements from the cells, thereby causing an increase in their concentrations. Moreover, the migration of metal elements from the cooking container into the food matrix may also result in increased concentrations of these elements in the final dishes. For instance, high-temperature cooking techniques, such as frying, tend to facilitate the dissolution of metallic elements into lipids (33). Furthermore, insufficient cleaning of cooking utensils prior to or following the cooking process may result in elemental residues, which could subsequently lead to increased elemental content in the final prepared dishes. In terms of elemental valence, the magnitude of increase in divalent metals (Mg, K, Ca, Mn, Cu, Zn) was notably higher compared to monovalent metals. This observation could be related to the enhanced capacity of divalent metal ions to form complexes with proteins (34). Likewise, the results of the inter-group comparative analysis indicated that, for both cooking modes, there were no statistically significant differences in the pre- and post-cooking changes in the content of the seven mineral elements in the dishes (*p* > 0.05).

## 4 Study strengths and limitations

As industrialized cuisine rapidly advances, the disparities in quality resulting from industrial cooking methods versus traditional hand-cooked techniques have increasingly garnered attention from both industry practitioners and consumers. In this study, we focused on the variations in nutrient content pre- and post-cooking resulting from the two distinct cooking methods, addressing a notable gap in existing research. In the present study, six industrially prepared Chinese cuisines commonly available in the Chinese market were selected for investigation. The primary evaluation metrics revolved around the content of essential macronutrients and micronutrients. We meticulously compared the changes in nutrient content changes pre- and post-cooking using both industrial and traditional hand-cooked modes, aiming to provide a comprehensive analysis of how these two cooking approaches impact the nutritional profile of the dishes. Additionally, across both cooking modes, the majority of nutrient levels exhibited consistent patterns of change pre- and post-cooking. In the case of the six dishes studied, the contents of water-soluble vitamins, dietary fiber, and sugars decreased to different extents after being cooked using both methods. On the other hand, the levels of proteins, fats, fatty acids, and fat-soluble vitamins experienced both declines and rises. After cooking under both modes, the mineral content in these dishes was observed to increase. Possible causes for the decrease in nutrient content were: (a) The myriad of chemical reactions occurring in foods during cooking, such as oxidation, thermal decomposition, photodecomposition, Maillard reactions, and caramelization. Carbohydrates predominantly engage in Maillard reactions with amino acids, yielding Maillard reaction products and caramel pigments through caramelization. High-temperature cooking further contributes to the dehydration and thermal degradation of sugars (35). (b) The loss of certain water-soluble nutrients during cooking due to moisture evaporation. Specifically, several water-soluble vitamins and elements can evaporate with water during steaming or boiling, subsequently condensing and draining to the bottom of the pot. Incomplete recovery of the broth leads to significant nutrient depletion.

Conversely, certain nutrients demonstrated varying degrees of increased content after cooking under both methods. Possible explanations for these observations could be: (a) The heating and stirring exacerbate the breakdown of raw material cells, leading to enhanced nutrient leaching (25). Furthermore, some nutrients, especially fat-soluble ones, are thermally stable, contributing to observed concentration increases. (b) The migration effect stemming from the cooking utensils utilized predominantly influences changes in elemental content (33). As all cooking tasks were executed using metal pots, a marked rise in iron and copper levels was observed across all samples post-cooking. Upon conducting inter-group *t*-tests for comparison, the changes in nutrient levels between the two cooking methods showed no statistically significant differences, with the exception of vitamin B6. This implied that there were negligible differences in nutrient content changes for the six dishes between the industrial and traditional hand-cooked modes. However, it is important to note that this study did not encompass all types of typical Chinese cuisine, cooking techniques, and nutrient categories. Consequently, whether the influence of industrial versus traditional hand-cooked methods on nutrient content changes in Chinese cuisine remains consistently similar across all scenarios necessitates further investigation. Additionally, while this study concentrated on the comparative analysis of major nutrient content changes, it is equally crucial to explore potential variations in nutrient composition and structural changes, which should be addressed in future research endeavors.

## 5 Conclusion

This study aimed to elucidate the differences in nutrient content alterations of Chinese cuisines subjected to industrial versus traditional hand-cooking modes. By analyzing six commonly available cuisines in the market, the research compared the changes in major nutrient contents before and after processing under the two cooking modes. The results indicated that, with the exception of a significantly greater reduction in vitamin B6 content in cuisines prepared via the traditional hand-cooking mode as opposed to the industrial mode, there were no significant differences in the changes of most nutrient contents between the two cooking modes (*p* > 0.05). However, this study did not encompass all representative categories of Chinese cuisines, cooking techniques, or nutrient types. Consequently, further research is necessary to ascertain whether the effects of industrial and traditional hand-cooking on nutrient content alterations in Chinese cuisines are consistently uniform. Additionally, this study concentrated solely on the changes in major nutrient contents, leaving potential differences in the compositional and structural transformations of nutrients to be explored in future investigations.

## Data Availability

The original contributions presented in the study are included in the article/Supplementary material, further inquiries can be directed to the corresponding authors.

## References

[1] Yu Q Zhang M Ju R. Advances in prepared dish processing using efficient physical fields: a review. Crit Rev Food Sci. (2024) 64:4031–45. doi: 10.1080/10408398.2022.2138260, PMID: 36300891

[2] Chen J Zhang Y Ren Y Chen X Feng Y Zhang Y. The formation mechanism and control strategies of warmed-over flavor in prepared dishes: a comprehensive review and future perspectives. Trends Food Sci Tech. (2024) 153:104746. doi: 10.1016/j.tifs.2024.104746

[3] Jia Y Hu L Liu R Yang W Khalifa I Bi J. Innovations and challenges in the production of prepared dishes based on central kitchen engineering: a review and future perspectives. Innov Food Sci Emerg. (2024) 91:103521. doi: 10.1016/j.ifset.2023.103521

[4] Lobefaro S Piciocchi C Luisi F Miraglia L Romito N Luneia R. Cooking techniques and nutritional quality of food: a comparison between traditional and innovative ways of cooking. Int J Gastron Food S. (2021) 25:100381. doi: 10.1016/j.ijgfs.2021.100381, PMID: 40046687

[5] Garg M Sharma A Vats S Tiwari V Kumari A Mishra V. Vitamins in cereals: a critical review of content, health effects, processing losses, bioaccessibility, fortification, and biofortification strategies for their improvement. Front Nutr. (2021) 8:586815. doi: 10.3389/fnut.2021.586815, PMID: 34222296 PMC8241910

[6] Lee S Choi Y Jeong HS Lee J Sung J. Effect of different cooking methods on the content of vitamins and true retention in selected vegetables. Food Sci Biotechnol. (2017) 27:333–42. doi: 10.1007/s10068-017-0281-1, PMID: 30263756 PMC6049644

[7] Li X Yu L Xie Y Li C Fang Z Hu B. Effect of different cooking methods on the nutrient, and subsequent bioaccessibility and biological activities in boletus auripes. Food Chem. (2023) 405:134358. doi: 10.1016/j.foodchem.2022.134358, PMID: 36370574

[8] Tang T Zhang M Law CL Mujumdar A. Novel strategies for controlling nitrite content in prepared dishes: current status, potential benefits, limitations and future challenges. Food Res Int. (2023) 170:112984. doi: 10.1016/j.foodres.2023.112984, PMID: 37316019

[9] Naomi NM Willis OO Jane A Daniel Sila N. Effect of drying methods on the retention of bioactive compounds in African eggplant. Food Sci Nutr. (2018) 6:814–23. doi: 10.1002/fsn3.623, PMID: 29983944 PMC6021694

[10] Jiangsu Provincial Standardization Administration (2009) General specifications for Huaiyang cuisine. DB32/T. 1548–2009. Nanjing: Jiangsu Provincial Quality and Technical Supervision Bureau.

[11] Sichuan Provincial Quality and Technical Supervision Bureau (2014) Specification for Chinese Sichuan cuisine cooking techniques. DB51/T. 1728–2014. Chengdu: Sichuan Provincial Quality and Technical Supervision Bureau.

[12] Guangzhou Municipal Market Supervision and Administration Bureau (2020) Specification for Guangfu cuisine cooking techniques. DB4401/T. 38–2020. Guangzhou: Guangzhou Municipal Market Supervision and Administration Bureau.

[13] Gunjan S Christine J Bandyopadhyay K Debarati P. Comparative analysis of biodiesel produced by acidic transesterification of lipid extracted from oleaginous yeast *Rhodosporidium toruloides*. Biotech. (2018) 8:434. doi: 10.1007/s13205-018-1467-9 PMC6170317 30306003

[14] Grażyna Ś Małgorzata J Jolanta K Norbert R Szymon Z Anna W. Influence of algae supplementation on the concentration of glutathione and the activity of glutathione enzymes in the mice liver and kidney. Nutrients. (2021) 13:1996. doi: 10.3390/nu13061996 34200606 PMC8227691

[15] Kim YA Park S Park Y Park GR Oh SG Choi J. Effect of addition of fermented soy sauce on quality characteristics of pork patties during refrigerated storage. Food Secur. (2022) 11:1004. doi: 10.3390/foods11071004, PMID: 35407090 PMC8997975

[16] Wu D Zhang L Zhang Y Shi J Tan P Zheng Z. Lipid profiles of human milk and infant formulas: a comparative lipidomics study. Food Secur. (2023) 12:600. doi: 10.3390/foods12030600, PMID: 36766129 PMC9914114

[17] Association of Official Analytical Chemists. Official Methods of Analysis [991.43]. Gaithersburg, MD: AOAC International‌ (2011).

[18] Liu Y Duan X Zhang M Li C Zhang Z Liu A. Cooking methods effect on the nutrients, bioaccessibility and antioxidant activity of *Craterellus cornucopioides*. LWT-Food Sci Technol. (2020) 131:109768. doi: 10.1016/j.lwt.2020.109768

[19] Tuncel NB Yılmaz N Kocabıyık H Uygur A. The effect of infrared stabilized rice bran substitution on B vitamins, minerals and phytic acid content of pan breads: part II. J Cereal Sci. (2014) 59:162–6. doi: 10.1016/j.jcs.2013.12.005

[20] Association of Official Analytical Chemists. Official Methods of Analysis [960.46]. Gaithersburg, MD: AOAC International‌ (2005).

[21] Rossi R Vizzarri F Ratti S Palazzo M Corino C. Effects of long-term supplementation with brown seaweeds and polyphenols in rabbit on meat quality parameters. Animals. (2020) 10:2443. doi: 10.3390/ani10122443, PMID: 33419317 PMC7766534

[22] Pei Z. Zhang L. Fang C. Yang J. Li J., and, Zhao Y. Wu Y. (2021). Assessment of dietary intakes of total fat and fatty acids for residents in China in 2015-2018. J Food Compos Anal 102,:104045. doi: 10.1016/j.jfca.2021.104045, PMID: 40046687

[23] Verma SK Ganesan P Kishore P Remya S Mohan C Padmavathy P. Effects of different cooking methods on the proximate composition and physical properties of Brown shrimp (*Metapenaeus dobsonii*) during cooking and freezing cycle. Food Sci Technol Int. (2024) 30:517–26. doi: 10.1177/10820132231166972 37041697

[24] Ng ZX Rosman NF. *In vitro* digestion and domestic cooking improved the total antioxidant activity and carbohydrate-digestive enzymes inhibitory potential of selected edible mushrooms. J Food Sci Tech Mys. (2019) 56:865–77. doi: 10.1007/s13197-018-3547-6, PMID: 30906044 PMC6400758

[25] Innosa D Ianni A Palazzo F Martino F Bennato F Grotta L. High temperature a-nd heating effect on the oxidative stability of dietary cholesterol in different real food syste-ms arising from eggs. Eur Food Res Technol. (2019) 245:1533–1538. doi: 10.1007/s00217-019-03266-4

[26] Yuan GF Sun B Liu ZQ Wang QM. Effects of different cooking methods on health-promoting compounds and antioxidant activity of broccoli. J Zhejiang Univ Sci B. (2009) 10:580–8. doi: 10.1631/jzus.B0920051, PMID: 19650196 PMC2722699

[27] Grundy MM Edwards CH Edwards AR Mackie AR Gidley MJ Peter R. Re-evaluation of the mechanisms of dietary fibre and implications for macronutrient bioaccessibility, digestion and postprandial metabolism. Br J Nutr. (2016) 116:816–33. doi: 10.1017/S0007114516002610, PMID: 27385119 PMC4983777

[28] Megido RC Poelaert C Ernens M Liotta M Blecker C Danthine S. Effect of household cooking techniques on the microbiological load and the nutritional quality of mealworms (*Tenebrio molitor* L. 1758). Food Res Int. (2018) 106:503–8. doi: 10.1016/j.foodres.2018.01.002, PMID: 29579954

[29] Liu K Zheng J Wang X Chen F. Effects of household cooking processes on mineral, vitamin b, and phytic acid contents and mineral bioaccessibility in rice. Food Chem. (2019) 280:59–64. doi: 10.1016/j.foodchem.2018.12.053, PMID: 30642507

[30] Aragão M Pires L Santos-Buelga C Barros L Calhelha RC. Revitalising riboflavin: unveiling its timeless significance in human physiology and health. Food Secur. (2024) 13:2255. doi: 10.3390/foods13142255, PMID: 39063339 PMC11276209

[31] Shewry PR Van Schaik F Ravel C Charmet G Rakszegi M Bedo Z. Genotype and environment effects on the contents of vitamins B1, B2, B3, and B6 in wheat grain. J Agric Food Chem. (2011) 59:10564–71. doi: 10.1021/jf202762b, PMID: 21863876

[32] Feng S Li P Li Y Xiao X Chen X Leng H. Volatile profiles and characteristic odorants in camellia seeds with different heat pretreatments. Food Chem. (2025) 468:142497. doi: 10.1016/j.foodchem.2024.142497, PMID: 39700800

[33] Llorent-Martinez EJ Ortega-Vidal J Ruiz-Riaguas A Ortega-Barrales P Fernandez-de Cordova ML. Comparative study of the phytochemical and mineral composition of fresh and cooked broccolini. Food Res Int. (2020) 129:108798. doi: 10.1016/j.foodres.2019.108798, PMID: 32036908

[34] Purchas RW Wilkinson BHP Carruthers F Jackson F. A comparison of the nutrient content of uncooked and cooked lean from New Zealand beef and lamb. J Food Compos Anal. (2014) 35:75–82. doi: 10.1016/j.jfca.2014.04.008

[35] Park H Seo H Cho IH. Effect of amino acids in the Maillard reaction products generated from the reaction flavors of *Tenebrio molitor* (mealworm) protein and d-xylose. Food Sci Biotechnol. (2022) 31:1647–60. doi: 10.1007/s10068-022-01158-0, PMID: 36312991 PMC9596655

