# Supplementary material for: A comparative analysis of nutritional content changes in six Chinese cuisines prepared using industrial versus traditional hand-cooked modes
Source: Front Nutr. 2025 Mar 19;12:1567196. doi: 10.3389/fnut.2025.1567196 (PMC11961429; doi:10.3389/fnut.2025.1567196)
Supplement: Supplementary file 1 [file Data_Sheet_1.docx]

**A Comparative Analysis of Nutritional Content Changes in Six Chinese** **Cuisines Prepared Using Industrial versus** **Traditional Hand-Cooked Modes**

Xuan Wang^1^, Jun Li^1^, Xiaomeng Wu^2^, Sai Fan^3^, Zhu Wang^4*^, Yunfeng Zhao^1^, Jingguang Li^1^, Dawei Chen^1*^

1 NHC Key Laboratory of Food Safety Risk Assessment, Food Safety Research Unit (2019RU014) of Chinese Academy of Medical Science, China National Center for Food Safety Risk Assessment, Beijing 100021, China

2 College of Food Science and Nutritional Engineering, China Agricultural University, Beijing 100083, China

3 Beijing center for disease prevention and control, Beijing 100013, China

4 National Institute for Nutrition and Health, Chinese Center for Disease Control and Prevention, Beijing 100050, China

* Correspondence:

Dr. Zhu Wang; Dr. Dawei Chen

wangzhu@ninh.chinacdc.cn (Z. Wang); chendw@cfsa.net.cn (D. Chen)

1 Chinese Cuisines preparation procedure

1.1 Braised Pork in Brown Sauce (BPBS)

A total of 500 g of pork was marinated with 10 g of ginger slices and 5 g of garlic. Subsequently, the mixture was combined with the following ingredients: 30 mL of soy sauce, 20 g of sugar, 15 mL of rice wine, 2 star anise pods, 1 cinnamon stick (weighing 2 g), and 2 bay leaves. The resultant mixture was then evenly divided into two portions. One portion was stewed to completion using industrial-grade steaming equipment, thereby exemplifying industrially processed BPBS, while the other portion was stewed using a conventional household steamer, serving as a representative example of traditional hand-cooked cuisine.

1.2 Braised Beef with Radish (BBR)

Five hundred grams of beef brisket were uniformly diced, and 300 grams of white radish were peeled and cut into pieces of equal size. These components were then combined with cooking oil, ginger slices, scallion strips, and one tablespoon each of rice wine, light soy sauce, and dark soy sauce. An adequate amount of water was added to ensure the meat was fully submerged. The mixture was subsequently divided into two equal portions. One portion was stewed to completion using industrial-grade steaming equipment, thus representing the industrially processed BBR. The other portion was stewed using a conventional household steamer, serving as an exemplar of traditional hand-cooked cuisines.

1.3 Steamed Pork with Preserved Vegetables (SPPV)

An initial 500 g of pork belly was boiled as a whole block until it reached approximately 70% doneness. Subsequently, the pork was marinated with one tablespoon each of soy sauce and dark soy sauce to enhance its color. The meat was then deep-fried in hot oil until its surface attained a golden-brown hue and a bubbly texture. Following this, the pork was sliced into pieces approximately 0.5 cm thick and carefully arranged at the base of a bowl. A total of 150 g of preserved mustard greens were soaked, cleaned, finely chopped, and mixed with minced garlic, sugar, and one tablespoon of light soy sauce, before being evenly distributed over the pork slices. The prepared dish was then divided into two equal portions: one portion was placed in an industrial-grade steamer and cooked to completion, resulting in an industrially prepared SPPV. The second portion was placed in a conventional household steamer and cooked to completion, representing a traditional hand-cooked cuisine.

1.4 Braised Tomato Beef (BTB)

Three hundred grams of beef were thinly sliced and marinated with a minimal amount of salt and one tablespoon of light soy sauce for a duration of 15 minutes. Two tomatoes were washed and cut into chunks. One tablespoon of cooking oil was introduced into the pan, followed by the addition of garlic cloves. A small supplementary amount of oil was then incorporated, and the tomato chunks were thoroughly mixed in. The preparation was subsequently divided into two portions: one portion was stir-fried in an industrial-grade wok until fully cooked, resulting in an industrially prepared BTB, while the other portion was stir-fried in a conventional household wok, yielding a traditionally hand-cooked cuisine.

1.5 Braised Beef with Potatoes (BBP)

Five hundred grams of beef were sectioned into uniformly sized pieces, while 300 grams of potatoes were peeled and cut into similarly sized segments. One tablespoon of cooking oil and soy sauce was incorporated into the mixture, followed by the addition of ginger slices, scallion shreds, and the potato pieces. An adequate amount of hot water was then introduced to ensure complete submersion of the beef. The mixture was subsequently divided into two equal portions: one portion was transferred to industrial-grade stewing equipment and cooked until fully prepared, thus exemplifying industrially processed BBP. The other portion was stewed using a conventional household steamer, serving as a representation of traditionally hand-cooked cuisines.

1.6 Braised Pork Ball in Brown Sauce (BPBBS)

A total of 500 grams of ground pork was prepared and combined with 100 grams of finely chopped rehydrated mushrooms, 50 grams of a scallion and ginger-infused liquid, one egg, two tablespoons of light soy sauce, and one teaspoon of salt. These ingredients were meticulously mixed and formed into approximately six large meatballs. In a preheated skillet, one tablespoon of cooking oil was added, and the meatballs were seared until their surfaces attained a golden-brown hue. Subsequently, three meatballs were transferred to industrial-grade stewing equipment for processing into industrially cooked BPBBS. The remaining three meatballs were placed in a traditional clay pot and slow-simmered over low heat to produce hand-cooked BPBBS, exemplifying traditional culinary techniques.
